# Integrating multi-omics and machine learning to reveal IL1R2-driven macrophage-fibroblast spatial interactions in polycyclic aromatic hydrocarbon-induced pulmonary fibrosis

**DOI:** 10.3389/fimmu.2026.1968775

**Published:** 2026-10-21

**Authors:** Xin Tang, Peiyi Bai, Qinyang Du, Wulin Yang, Gaopeng Li

**Affiliations:** 1 Hefei Cancer Hospital of Chinese Academy of Sciences (CAS), Institute of Health and Medical Technology, Hefei Institutes of Physical Science, Chinese Academy of Sciences, Hefei, China; 2 Science Island Branch, Graduate School of University of Science and Technology of China, Hefei, China; 3 Department of General Surgery (Hepatobiliary Surgery), Third Hospital of Shanxi Medical University, Shanxi Bethune Hospital, Shanxi Academy of Medical Sciences, Tongji Shanxi Hospital, Taiyuan, China; 4 Burns Department, General Hospital of Taiyuan Iron and Steel Group Co., Ltd; (Sixth Hospital of Shanxi Medical University), Taiyuan, China

**Keywords:** idiopathic pulmonary fibrosis, IL1R2, molecular docking, network toxicology, polycyclic aromatic hydrocarbons, single-cell transcriptomics

## Abstract

**Background:**

Polycyclic aromatic hydrocarbons (PAHs) are ubiquitous environmental toxicants, yet the toxicological mechanisms by which they induce idiopathic pulmonary fibrosis (IPF) remain poorly understood. This study aims to systematically elucidate the molecular basis and cellular interplay driving PAH-induced pulmonary fibrosis.

**Methods:**

We first evaluated the toxicological profiles of 16 priority-controlled PAHs using toxicological databases and then conducted *in vitro* experiments to assess their effects on pulmonary epithelial cells. By integrating transcriptomic data, we combined differential expression analysis, weighted gene co-expression network analysis (WGCNA), and Mendelian randomization (MR) to screen IPF-associated genes. Given the well-defined structure of IL1R2, molecular docking and molecular dynamics (MD) simulations were employed to evaluate the binding affinity and conformational stability of the 16 PAHs with IL1R2. Finally, using single-cell transcriptomics, we compared the functional disparities between IL1R2-positive and IL1R2-negative macrophages within the fibrotic microenvironment.

**Results:**

*In vitro*, PAHs exerted cytotoxic effects on pulmonary epithelial cells, manifested primarily as suppressed cell viability, dysregulated inflammatory cytokine expression, and DNA damage. Intersecting the identified IPF-associated genes with PAH-target genes from the CTD database yielded diagnostic and prognostic models, ultimately pinpointing SPP1, IL1R2, and SCGB3A2 as core targets. Molecular docking and MD simulations demonstrated stable binding of the 16 PAHs to IL1R2. Single-cell transcriptomic analysis further revealed that IL1R2-negative macrophages mediate a more pronounced pro-fibrotic phenotype via the IL-1 signaling pathway and exacerbate pro-fibrotic cell–cell communication with fibroblasts.

**Conclusions:**

In summary, this study clarifies the key targets and cellular mechanisms underlying PAH-mediated respiratory toxicity, providing a multidimensional research strategy for the risk assessment of environmental pollutant-induced pulmonary fibrosis.

## 1 Introduction

Polycyclic aromatic hydrocarbons (PAHs) are a class of organic compounds consisting of two or more fused benzene rings, primarily originating from the incomplete combustion of fossil fuels and biomass (1, 2). As persistent environmental toxicants ubiquitous in the atmosphere, water, and soil, PAHs exhibit high lipid solubility and resistance to degradation. Upon entering the respiratory tract, they readily inflict continuous damage on pulmonary tissues (3, 4).

In recent years, the potential association between PAH exposure and idiopathic pulmonary fibrosis (IPF) has garnered increasing attention. IPF is characterized by alveolar epithelial micro-injury and aberrant tissue repair, pathological processes that can be triggered and amplified by the toxic effects of PAHs. Epidemiological studies have demonstrated that elevated levels of urinary PAH metabolites are significantly associated with a substantial decline in lung function (5), highlighting the destructive impact of PAH exposure on the lung parenchyma. Mechanistically, upon entering the alveolar space, PAHs not only directly compromise the alveolar epithelial barrier but also disrupt the local immune microenvironment. Low-molecular-weight PAHs (e.g., phenanthrene and fluorene) can induce profound oxidative stress and inflammatory responses in human bronchial epithelial cells (6, 7). Such toxicant-induced epithelial injury and oxidative imbalance represent key initiating events in the pathogenesis of IPF. Furthermore, PAHs can mediate the release of pro-inflammatory cytokines and the aberrant activation of macrophages (8–10), thereby further driving aberrant tissue remodeling. In contrast, high-molecular-weight PAHs (e.g., benzo[a]pyrene) exhibit stronger lipophilicity, allowing them to persist longer within pulmonary tissues. Their reactive metabolites (e.g., diol epoxides) can bind to DNA to form adducts, leading to cellular mutations and interfering with normal cellular metabolism and gene expression (11, 12), consequently exacerbating chronic pathological damage in the lung. Notably, the impact of PAHs on pulmonary health can be traced back to early developmental stages. Studies indicate that maternal exposure to PAHs can exacerbate hyperoxia-induced lung injury in neonates (13). Such early-life exposure may alter pulmonary developmental programming, thereby increasing an individual’s subsequent susceptibility to toxicant-induced fibrosis.

Given the inherent limitations of relying solely on network toxicology predictions to construct complex pathogenic networks, this study integrates multi-omics analyses, computational chemistry simulations, and *in vitro* cellular assays to systematically elucidate the toxicological mechanisms underlying PAH-induced IPF. Initially, we evaluated the cytotoxicity and DNA damage potential of 16 priority-controlled PAHs. Subsequently, by integrating weighted gene co-expression network analysis (WGCNA) and Mendelian randomization (MR), we screened IPF-associated genes and intersected them with PAH toxicological targets from the CTD database to construct diagnostic and prognostic models. This pipeline ultimately pinpointed SPP1, IL1R2, and SCGB3A2 as core targets. Focusing on IL1R2, molecular docking and molecular dynamics (MD) simulations were employed to verify the conformational stability and high binding affinity of PAHs to this receptor. Finally, leveraging single-cell and spatial transcriptomic data, we revealed the functional remodeling of IL1R2-negative macrophages within the fibrotic microenvironment and characterized their spatial intercellular communication network with fibroblasts. This study aims to clarify the molecular basis and cellular interplay driving PAH-induced pulmonary toxicity, thereby providing multidimensional scientific evidence for the risk assessment and clinical intervention of environmental pollutant-related pulmonary fibrosis.

## 2 Materials and methods

### 2.1 Toxicity prediction and analysis of PAHs

To systematically assess the toxicological profiles of polycyclic aromatic hydrocarbons (PAHs), we retrieved the structural data of 16 representative PAHs from the PubChem database and predicted their toxicity using the ADMETlab 3.0 platform (14). The selected target compounds included naphthalene, acenaphthylene, acenaphthene, fluorene, phenanthrene, anthracene, fluoranthene, pyrene, benz[a]anthracene, chrysene, benzo[b]fluoranthene, benzo[k]fluoranthene, benzo[a]pyrene, indeno[1,2,3-cd]pyrene, dibenz[a,h]anthracene, and benzo[ghi]perylene. These substances are ubiquitous in the environment, highly persistent, and prone to bioaccumulation. Given their close association with human exposure and related health risks, they are widely designated as priority pollutants under international regulatory frameworks.

### 2.2 Construction of WGCNA and identification of disease-associated modules

The IPF transcriptomic datasets utilized in this study (GSE110147 (15), GSE32537 (16), GSE10667 (17), GSE47460 (18), GSE48149 (19), and GSE53845 (20))were retrieved from the Gene Expression Omnibus (GEO) database. Datasets GSE110147 and GSE32537 were merged to serve as the training set, while the remaining datasets were designated as the validation set. To identify IPF-associated hub genes, a weighted gene co-expression network analysis (WGCNA) was constructed based on the training set. For the analysis, genes exhibiting the top 50% variance in expression were selected as input data. After determining the appropriate soft threshold, an adjacency matrix was constructed, followed by hierarchical clustering. Subsequently, Pearson correlation analysis was employed to evaluate the strength of the association between module eigengenes and the IPF clinical phenotype. Modules demonstrating significant correlations were defined as core modules, and the genes within these modules were extracted for the subsequent construction of the disease target repository.

### 2.3 Screening of targets associated with PAHs and IPF

To identify potential targets involved in PAH-induced IPF, we systematically queried the Comparative Toxicogenomics Database (CTD, https://ctdbase.org/) using “Polycyclic Aromatic Hydrocarbons” as the keyword to retrieve PAH-related targets. Subsequently, the retrieved targets were integrated and deduplicated with the IPF-associated module genes identified via WGCNA to construct a unified disease target repository.

### 2.4 Pathway enrichment analysis

To elucidate the biological functions and associated signaling pathways of the core targets, functional enrichment analysis was performed utilizing the Kyoto Encyclopedia of Genes and Genomes (KEGG, https://www.genome.jp/kegg/) (21) and Gene Ontology (GO, https://geneontology.org) (22) databases. The thresholds for statistical significance were set at *p* < 0.05 and false discovery rate (FDR) < 0.05.

### 2.5 Mendelian randomization analysis

Two-sample Mendelian randomization (MR) analysis was performed using the TwoSampleMR package (23). Single nucleotide polymorphisms (SNPs) reaching the genome-wide significance level (*P* < 5×10^-8^) were selected as instrumental variables (IVs), and those associated with potential confounders were removed. To ensure the independence of the IVs, linkage disequilibrium (LD) clumping was performed (*R*² < 0.01, window size of 10,000 kb). Exposure data were obtained from expression quantitative trait loci (eQTLs) in the IEU OpenGWAS database, while outcome data were derived from the idiopathic pulmonary fibrosis (IPF) cohort (ID: finn-b-IPF) within the same database. The inverse variance weighted (IVW) method was primarily used to estimate causal effects, with a significance threshold set at *P* < 0.05. The *F*-statistic was calculated to assess the strength of the association between the IVs and the exposure, and weak instruments with an *F*-statistic < 10 were excluded. Heterogeneity was evaluated using Cochran’s *Q* test, and a leave-one-out sensitivity analysis was conducted to verify the robustness of the results. The MR-Egger regression intercept was used to test for horizontal pleiotropy, while the MR-PRESSO method was applied to detect and correct for potential pleiotropic bias. The results of these tests are detailed in the **Supplementary Materials**.

### 2.6 Molecular docking

To validate the interactions between PAHs and the core target proteins and assess their binding affinities, molecular docking analysis was performed in this study. Given the absence of experimentally resolved crystal structures for SPP1 and SCGB3A2, docking experiments were conducted exclusively for the IL1R2 protein (24). The SDF structure files of the 16 PAH ligands were retrieved from the PubChem database, while the receptor structure of the IL1R2 protein was downloaded from the PDB database. During receptor preprocessing, water molecules, ions, and the co-crystallized ligand were removed from the native structure using PyMOL software, followed by format conversion and the addition of hydrogen atoms via Open Babel.

To ensure the accuracy of binding mode predictions and the reliability of affinity assessments, two docking algorithms, AutoDock Vina (25) and DeepMice (26), were employed. Specifically, AutoDock Vina rapidly evaluates the ligand-receptor binding energy based on a traditional empirical scoring function, whereas DeepMice, a deep learning-based tool, leverages neural networks to more precisely predict the binding modes of ligand-receptor complexes. During the docking process, a blind docking strategy covering the entire protein surface was applied to the IL1R2 protein to identify the optimal binding site. Following docking, the PLIP tool (27) was utilized to analyze the types of interactions and the key amino acid residues between the ligand and receptor. Additionally, Discovery Studio software was employed to visualize the three-dimensional binding modes of the PAH-protein complexes.

### 2.7 Molecular dynamics simulation

To verify the binding stability of the DBA-IL1R2 complex, a representative complex exhibiting the strongest binding energy was selected for a 100 ns molecular dynamics (MD) simulation. The simulation was performed using GROMACS 2024.2 (28). The AMBER99SB-ILDN force field was applied to the protein, while the ligand topology was generated using the ACPYPE program under the GAFF force field. The complex was placed in a periodic cubic water box with a minimum edge distance of 1.0 nm, solvated using the SPC/E water model, and Na^+^ ions were added to neutralize the system charge. Prior to the simulation, energy minimization was performed using the steepest descent method to eliminate steric hindrances, with a convergence criterion set at 1000 kJ·mol^-^¹·nm^-^¹. Subsequently, 100 ps NVT and 100 ps NPT ensemble equilibrations were performed sequentially. The temperature and pressure were maintained at 300 K and 1 bar using the V-rescale and Parrinello-Rahman algorithms, respectively. Long-range electrostatic interactions were treated using the PME method, and the cutoff radius for both van der Waals and short-range electrostatic interactions was set to 1.0 nm. The LINCS algorithm was used to constrain bonds involving hydrogen, with an integration step of 2 fs. Finally, a 100 ns production simulation was conducted.

### 2.8 Single-cell transcriptomic data processing

The single-cell transcriptomic data were obtained from the GSE136831 dataset in the GEO database (29). We utilized the Seurat package (30) to perform standard quality control and normalization, and strictly followed the parameter settings provided in the original study to execute UMAP dimensionality reduction, clustering analysis, and cell annotation. Following the identification of cell subpopulations, CellChat was further applied to comprehensively analyze the cell-cell communication networks among different cell populations. Furthermore, to evaluate the activity of specific biological pathways at the single-cell level, the AddModuleScore function was employed to calculate the IL-1 signaling scores and fibrosis scores for each cell subpopulation, aiming to elucidate the remodeling mechanisms of the pro-fibrotic microenvironment.

To validate the *in situ* physical spatial distribution of the aforementioned cell-cell interactions, spatial transcriptomic data from IPF and control groups were integrated (31). Human IPF spatial transcriptomics data were obtained from ArrayExpress (accession number E-MTAB-14121), comprising 4 healthy control and 4 IPF patient samples. The Seurat package was utilized to import the standard output files from 10x Visium Space Ranger and perform log-normalization. To evaluate the spatial abundance of macrophages and fibroblasts, module scores were calculated using the AddModuleScore function based on specific marker gene sets (macrophages: CD68, C1QA, C1QB, LYZ, AIF1; fibroblasts: ACTA2, COL1A1, COL1A2, DCN, PDGFRa) with parameters set as nbin = 10 and ctrl = 50. Subsequently, spatial spots with both Mac_Score and Fibro_Score in the top 25% (above the 75th percentile) were defined as macrophage-fibroblast co-localized regions. This approach enabled us to elucidate the structural basis of paracrine communication mediated by IL1R2 loss at the *in situ* tissue level.

### 2.9 Construction of IPF prognostic and diagnostic models

The GSE70866 dataset was used for prognostic model construction, comprising 176 IPF samples and 21 control samples (32). To construct the IPF prognostic risk model, the dataset was randomly divided into a training set and a validation set at a ratio of 7:3. Subsequently, the 9 screened target genes were subjected to LASSO Cox regression analysis (using the “glmnet” package in R) based on the training set. The optimal penalty parameter (λ) was determined via 10-fold cross-validation, and the risk score for each patient was calculated. First, multivariate Cox regression analysis was used to determine whether the risk score is an independent prognostic factor for IPF, controlling for age, gender, and Gender, Age, Physiology (GAP) stage. Based on the median risk score, patients were stratified into high- and low-risk groups. Survival curves were plotted using the Kaplan-Meier method, and survival differences between the groups were compared using the log-rank test. This analysis was performed separately in the training and validation sets. For IL1R2, SCGB3A2, and SPP1, independent prognostic survival analyses were conducted, with patients grouped according to the median expression levels of each gene. Time-dependent receiver operating characteristic (ROC) curves were utilized to evaluate the performance of the prognostic model in predicting 1- and 3-year survival, and the area under the curve (AUC) was calculated. For the construction of the diagnostic model, an IPF diagnostic classifier was built by combining LASSO regression and the XGBoost machine learning algorithm based on the expression data of IL1R2, SCGB3A2, and SPP1. To validate the robustness of the diagnostic model, the AUC values were calculated across 6 independent IPF cohorts and then averaged. Additionally, an independent ROC analysis was performed for SPP1 to evaluate its performance as a single-gene diagnostic biomarker in distinguishing IPF patients from controls.

### 2.10 Cell culture and PAHs cytotoxicity assay

Human bronchial epithelial cells (A549 and BEAS-2B cells, kindly provided by Professor Yannan Chu’s research group at the Hefei Institutes of Physical Science, Chinese Academy of Sciences) were cultured in a humidified incubator at 37 °C with 5% CO_2_. The culture medium used was DMEM (Gibco, USA) supplemented with 10% fetal bovine serum (FBS, Gibco, USA). Four PAH molecules, Dibenz[a,h]anthracene (Cat#: SJ-MX11233, SparkJade, China), Indeno[1,2,3-cd]pyrene (Cat#: SJ-MX11232, SparkJade, China), Phenanthrene (Cat#: HY-B1727, MCE, USA), and Benzo[a]pyrene (Cat#: SJ-MM0032, SparkJade, China), were prepared as solutions using DMSO. Cell viability was assessed using the Cell Counting Kit-8 (CCK-8) assay kit (Cat#: C0005, TargetMol, China). Target cells were pre-seeded into 96-well plates at a density of 3,000 cells per well. After 24 hours, the PAH solutions were added, and the cells were further cultured for 24 h or 48 h. Subsequently, 10 μL of CCK-8 solution was added to each well, followed by incubation at 37 °C for 1 hour. The substrate absorbance (OD450) was measured at a wavelength of 450 nm using a microplate reader, and the relative cell viability was calculated. All cell viability assays were performed in three independent biological replicates (n=3), each with three technical replicates. The selection of these four compounds was based on two main considerations: (1) Binding affinity: Molecular docking indicated that DBA exhibited the lowest binding energy and that INP also showed strong affinity, ranking among the top-scoring PAHs and predicting the strongest binding interactions with the IL1R2 receptor; (2) Ring number and toxicity representativeness: To cover a spectrum of structural complexity and toxicological potency, we included the 3-ring Phe as a representative of low-molecular-weight (LMW) PAHs with lower affinity. Conversely, BaP (5-ring), DBA (5-ring), and INP (6-ring) were selected to represent high-molecular-weight (HMW) PAHs known for their high carcinogenic potential.

### 2.11 Real-time quantitative PCR

Total cellular RNA was extracted using the TRIzol™ reagent (Cat#: SM130, Seven, China). After reverse transcription into cDNA using the SevenClever™ First Strand cDNA Synthesis Kit (Cat#: SM136, Seven, China), real-time quantitative PCR was performed on a HealForce X960 thermal cycler using the 2× SYBR Green qPCR MasterMixII premix (Cat#: SM143, Seven, China). The reaction program was set as follows: initial denaturation at 95 °C for 30 seconds, followed by 40 cycles of amplification (each cycle consisted of denaturation at 95 °C for 30 seconds, annealing at 60 °C for 10 seconds, and extension at 72 °C for 20 seconds). The relative mRNA expression levels of the target genes were measured using the 2-ΔΔCt method. All qPCR experiments were performed in three independent biological replicates (n=3), each with three technical replicates. Primer sequences are provided in the **Supplementary Materials**.

### 2.12 Macrophage and fibroblast co-culture

To validate the impact of PAH exposure on macrophage-driven fibroblast activation, an *in vitro* macrophage-fibroblast co-culture model was established. Briefly, THP-1 cells (Cat#: H3-0901, Cyagen, China) were differentiated into macrophages by treatment with 100 ng/mL phorbol 12-myristate 13-acetate (PMA, Cat#: TQ0198, TargetMol, China) for 24 h. Subsequently, the macrophages were exposed to dibenz[a,h]anthracene (DBA) and indeno[1,2,3-cd]pyrene (INP) at a concentration of 10 μM for 24 h. For the co-culture assay, a Transwell non-contact system (0.4 μm pore size, Corning, USA) was utilized. MRC-5 human lung fibroblasts (Cat#: AC178, Abcells, China) were seeded into the lower chamber at a density of 2 × 10^5^ cells/well. Upon adherence, the PAH-treated or DMSO-vehicle control macrophages were seeded into the upper chamber at a density of 5 × 10^4^ cells/well. After 48 h of co-culture, the MRC-5 cells in the lower chamber were harvested, and the expression level of the fibroblast activation marker α-SMA (Cat#: A33150, Nature Biosciences, China) was evaluated by Western blot. The co-culture experiments were independently repeated three times (n = 3).

### 2.13 Statistical analysis

Data mining, statistical analysis, and visualization in this study were implemented within the Python 3.9 and R 4.1 software environments. For data visualization, R packages including ggplot2, ComplexHeatmap, and ggpubr were primarily utilized to generate high-quality figures, encompassing gene expression distribution plots, survival analysis curves, and cell-cell communication networks. For statistical analysis, the Shapiro-Wilk test was first applied to assess the normality of the data. For data following a normal distribution with homogeneity of variance, comparisons between two groups were performed using the independent samples *t*-test, while comparisons among multiple groups were conducted using one-way analysis of variance (ANOVA). For data that did not meet the normality assumption or exhibited heterogeneous variances, the Mann-Whitney *U* test or the Kruskal-Wallis test was employed, respectively. Additionally, correlation analyses were performed using either the Pearson or Spearman correlation coefficient based on the data distribution characteristics. All statistical tests were two-sided, and a *P* value < 0.05 was considered statistically significant.

## 3 Results

### 3.1 Physicochemical properties and environmental toxicity assessment of PAHs

We first profiled the physicochemical properties and environmental toxicity of 16 representative PAHs via ADMET prediction (**Figure 1A**). As shown in **Figure 1B**, molecular weight positively correlated with lipophilicity (logP), but inversely correlated with the bioconcentration factor (BCF). This increase in logP also mirrored the rising median lethal concentration (LC_50_) in both *Pimephales promelas* and *Xenopsylla cheopis*. Notably, all 16 PAHs exhibited high toxic potential, particularly regarding eye irritation, genotoxicity, and respiratory toxicity (**Figure 1C**; full data in **Supplementary Materials**). Given their highly similar toxicity profiles, we hypothesized a common toxicological mechanism among these PAHs, providing a rationale for further exploring their targets in respiratory diseases.

**Figure 1:**
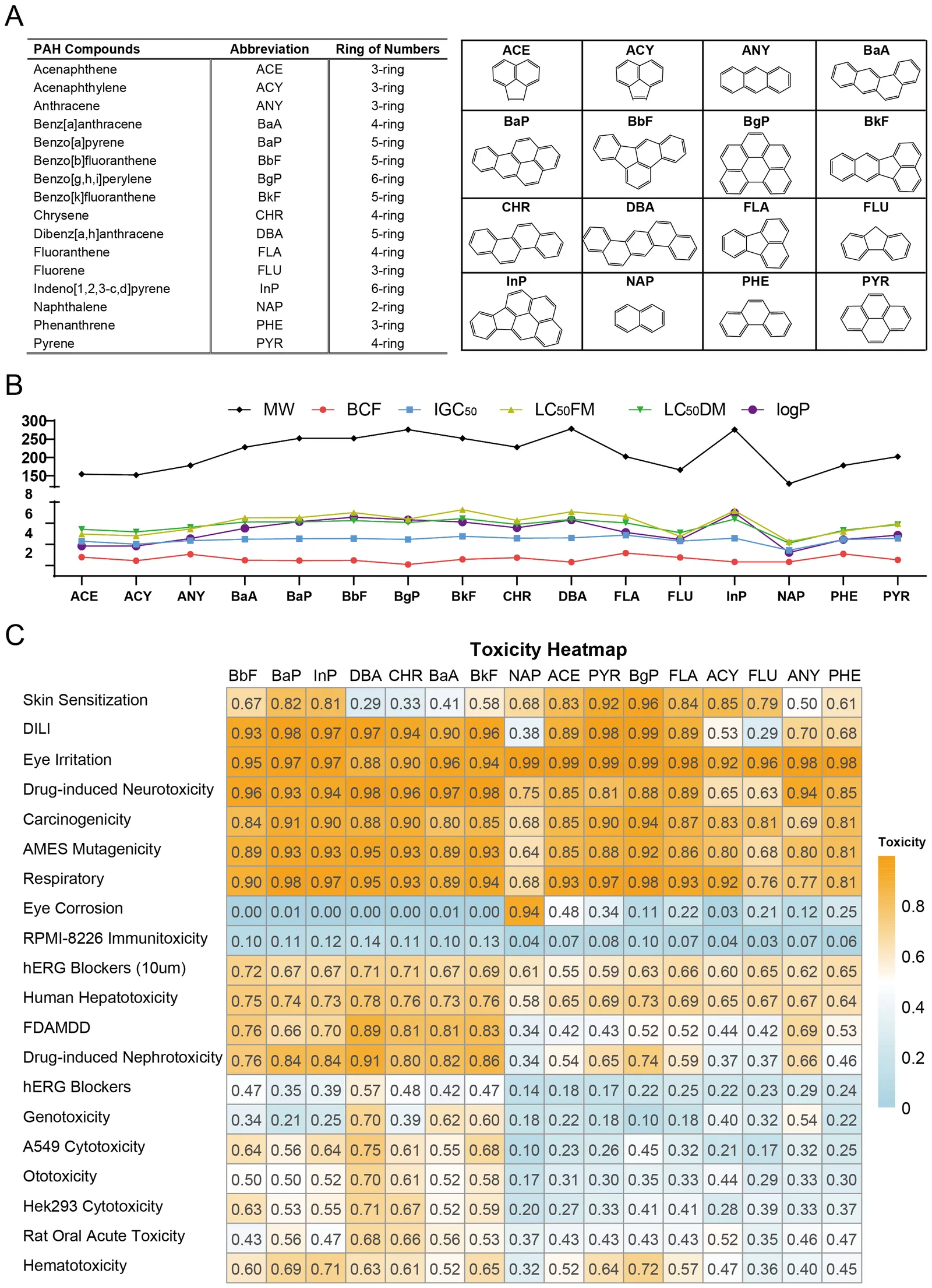
Physicochemical properties and toxicity profiles of 16 PAHs. **(A)** Chemical structures of the 16 PAHs. **(B)** Physicochemical properties and environmental toxicity metrics, including molecular weight (MW), octanol-water partition coefficient (log P), bioconcentration factor (BCF), and species-specific toxicity endpoints: LC_50_DM (*Xenopsylla cheopis*, 48 h), LC_50_FM (*Pimephales promelas*, 96 h), and IGC_50_ (*Tenebrio molitor*). **(C)** Predicted toxicity profiles based on ADMET analysis. Color intensity and numerical values indicate relative toxicity strength (higher values represent greater toxicity).

### 3.2 Effects of PAH exposure on cytotoxicity and inflammatory cytokine expression in bronchial/lung epithelial cells

We next evaluated the cytotoxicity of Indeno[1,2,3-c,d]pyrene, Dibenz[a,h]anthracene, Phenanthrene, and Benzo[a]pyrene in BEAS-2B and A549 cells. Using a CCK-8 assay across a concentration gradient (0–32 μM) for 24 and 48 hours (**Figures 2A–H**), we observed distinct cell type- and time-dependent responses. Indeno[1,2,3-c,d]pyrene and Benzo[a]pyrene significantly reduced viability in both cell lines at 32 μM after 24 h, with further suppression at 48 h. Conversely, Dibenz[a,h]anthracene and Phenanthrene exhibited cell type-specific toxicity; only A549 cells showed significant viability loss at 32 μM after 24 h, whereas BEAS-2B cells remained unaffected.

**Figure 2:**
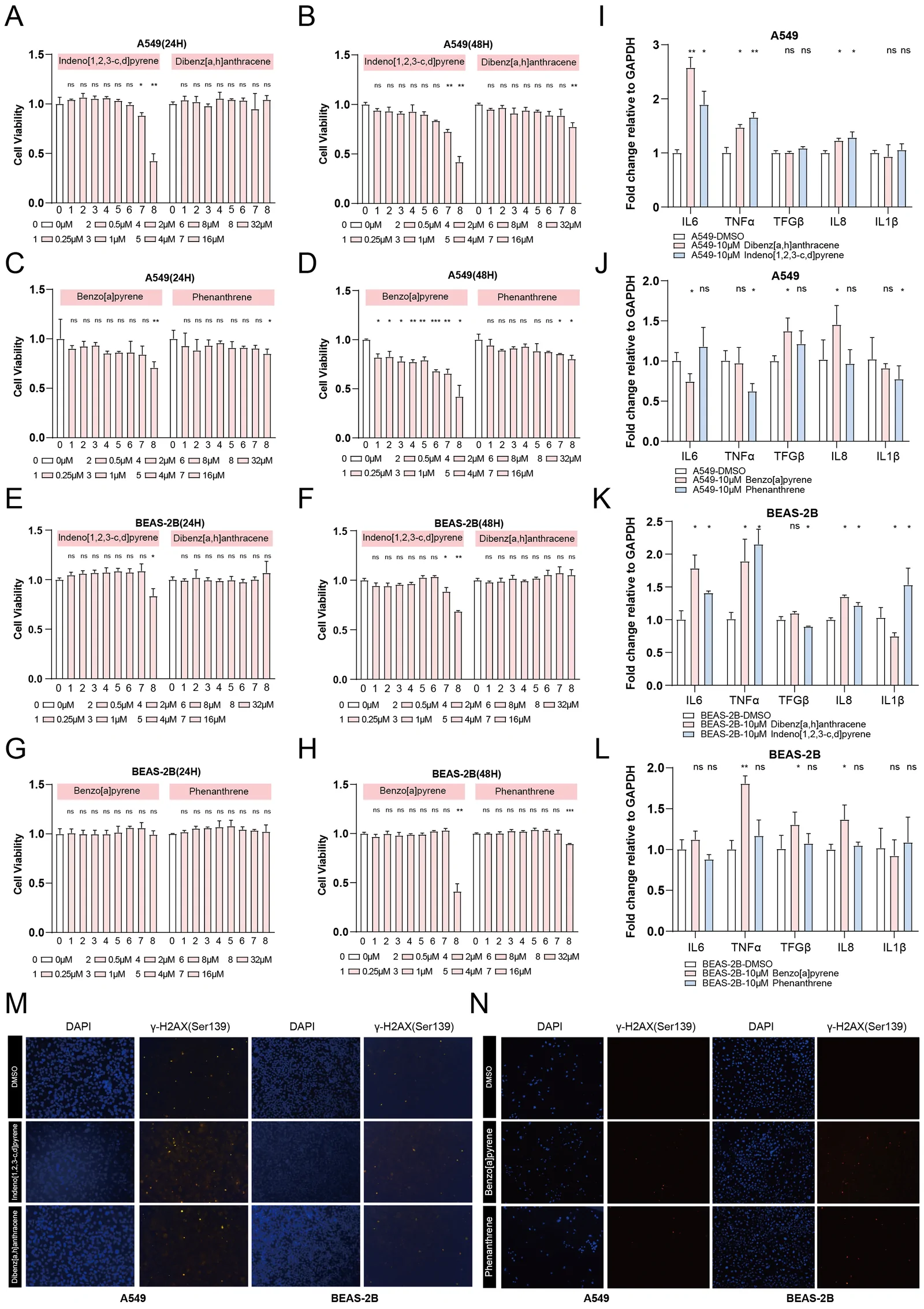
PAH-induced cytotoxicity, inflammatory response, and DNA damage in A549 and BEAS-2B cells. **(A–H)** Cell viability analysis. A549 **(A–D)** and BEAS-2B **(E–H)** cells were exposed to increasing concentrations (0–32 μM) of Dibenz[a,h]anthracene, Indeno[1,2,3-c,d]pyrene, Phenanthrene, or Benzo[a]pyrene for 24 h **(A, C, E, G)** and 48 h **(B, D, F, H)**, measured by CCK-8 assay. **(I–L)** qPCR analysis of inflammatory cytokines. Relative mRNA expression levels of IL6, TNFα, TGFβ, IL8, and IL1β (normalized to GAPDH) in A549 **(I, J)** and BEAS-2B **(K, L)** cells following a 24 h treatment with 10 μM of the indicated PAHs. **(M, N)** DNA damage assessment. Immunofluorescence staining of γ-H2AX (Ser139) in A549 and BEAS-2B cells treated with 20 μM PAHs for 48 h. DAPI was used for nuclear counterstaining, and DMSO served as the vehicle control. *P < 0.05, **P < 0.01; ns, not significant. Data are presented as mean ± SD from three independent biological replicates (n = 3). Comparisons among multiple groups were analyzed by one-way ANOVA followed by Dunnett’s *post hoc* test.

To assess inflammatory responses without confounding cell death, we treated cells with a sub-toxic dose (10 μM) for 24 h and measured cytokine mRNA levels via qPCR (**Figures 2I–L**). In BEAS-2B cells, Dibenz[a,h]anthracene, Indeno[1,2,3-c,d]pyrene, and Benzo[a]pyrene significantly upregulated IL-6, TNFα, and IL-1β. A549 cells showed a similar but slightly varied response: Dibenz[a,h]anthracene and Indeno[1,2,3-c,d]pyrene compounds increased IL-6 and TNFα, while Indeno[1,2,3-c,d]pyrene also induced IL-1β.

DNA damage was assessed by γ-H2AX immunofluorescence. Following 48 h of exposure to 20 μM of each compound, all four PAHs enhanced the γ-H2AX signal in A549 cells, with Dibenz[a,h]anthracene exhibiting the strongest, dose-dependent induction. In BEAS-2B cells, Dibenz[a,h]anthracene, Phenanthrene, and Benzo[a]pyrene caused pronounced DNA damage, whereas Indeno[1,2,3-c,d]pyrene yielded only a mild increase, consistent with the viability assay results (**Figures 2M, N**). Overall, these findings indicate that key PAHs induce epithelial cytotoxicity and DNA damage while selectively activating pro-inflammatory cytokines in the respiratory microenvironment.

### 3.3 Identification of hub genes associated with IPF

To systematically identify IPF-related hub genes, we first merged the GSE110147 and GSE32537 transcriptomic datasets and corrected for batch effects (**Figure 3A**). Subsequently, differentially expressed genes (DEGs) associated with IPF were screened through differential expression analysis (**Figures 3B, C**). Based on this, a gene co-expression network was constructed using Weighted Gene Co-expression Network Analysis (WGCNA) (**Figures 3D–F**), identifying a total of 5 co-expression modules (**Figure 3G**). Module-trait association analysis indicated that the blue, brown, and turquoise modules were significantly correlated with the IPF phenotype (**Figure 3G**). By evaluating module membership (MM) and gene significance (GS) (**Figures 3H, I**), we extracted the hub genes within the aforementioned key modules and intersected them with the DEGs. Ultimately, 403 overlapping genes were obtained and designated as potential hub genes driving the occurrence and development of IPF (**Figure 3J**).

**Figure 3:**
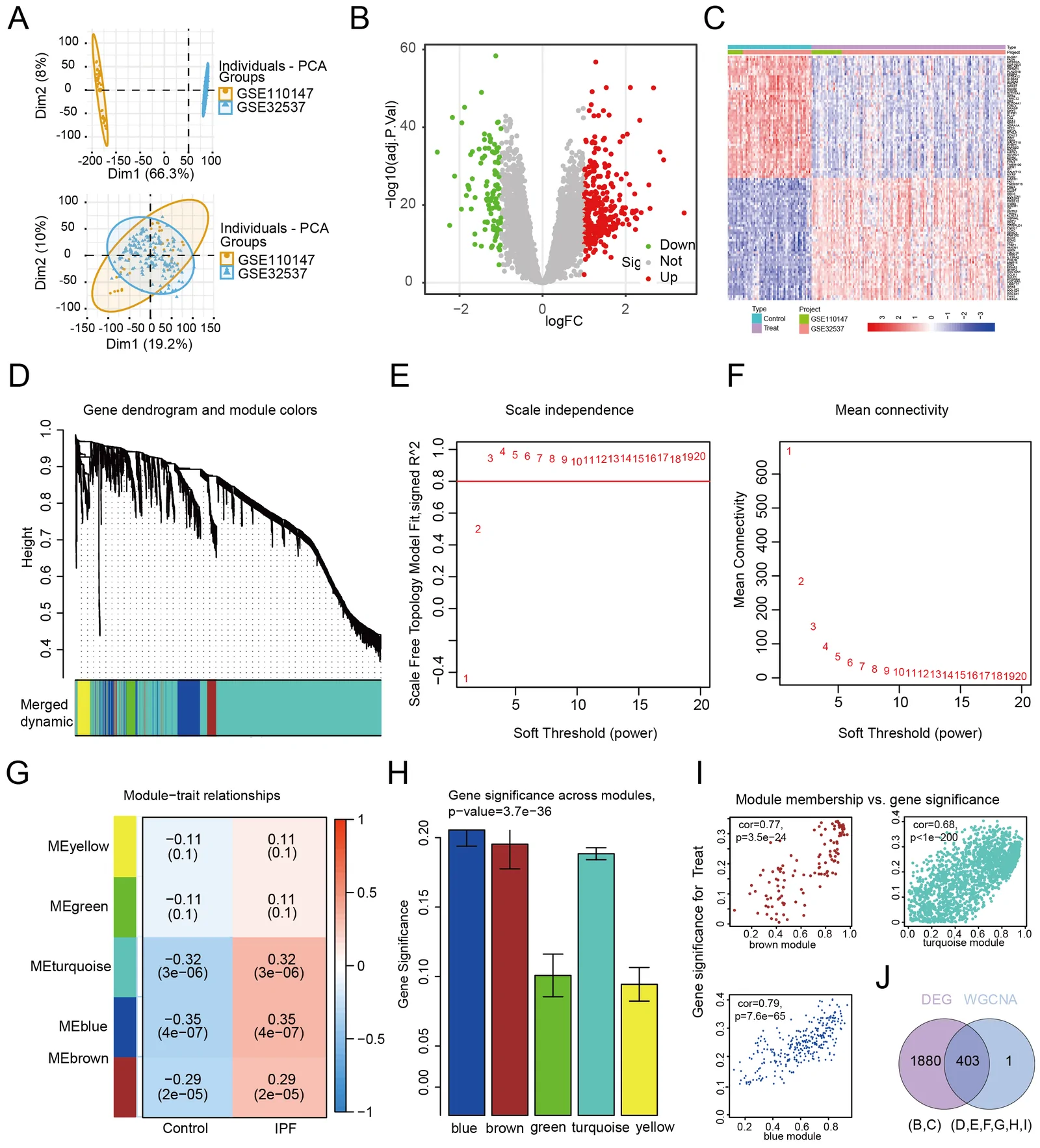
Identification of IPF-related genes. **(A)** Principal component analysis (PCA) plots before and after batch effect correction. **(B)** Volcano plot of differentially expressed genes. Red, blue, and gray dots represent genes significantly upregulated, downregulated, and not significantly different in the IPF group, respectively. **(C)** Heatmap of DEG expression in the control and IPF groups. **(D)** WGCNA gene clustering dendrogram. Hierarchical clustering based on gene co-expression levels, with branch colors representing different co-expression modules. **(E, F)** Selection of soft thresholding parameters. When the soft thresholding power (β) was set to 7, the scale-free topology fit index (*R*²) was > 0.85. **(E)** Scale independence. **(F)** Mean connectivity. **(G)** Heatmap of the association between WGCNA modules and the IPF clinical phenotype. **(H)** Distribution of gene significance (GS) across modules. **(I)** Scatter plots showing the correlation between gene significance (GS) and module membership (MM) in the blue, brown, and turquoise modules. **(J)** Venn diagram of DEGs and genes from key WGCNA modules.

### 3.4 Identification of shared targets between PAHs and IPF via Mendelian randomization

By intersecting the aforementioned 403 IPF-related genes with PAH target genes from the CTD database, a total of 109 overlapping genes were obtained (**Figure 4A**). Subsequently, using the eQTL and pQTL data of these 109 genes as instrumental variables, a two-sample Mendelian randomization (MR) analysis was conducted to evaluate their causal associations with IPF. The results revealed that four target genes (VCAM1, IL1R2, COL6A3, and CLCA2) were screened based on pQTL data (**Figure 4B**), and five target genes (SPP1, SCGB3A2, RTKN2, HSD17B13, and ADAM28) were identified based on eQTL data (**Figure 4C**). A heatmap visually illustrates the expression differences of these target genes between the control and IPF groups (**Figure 4B**). Furthermore, GO and KEGG enrichment analyses indicated that these 109 overlapping genes were primarily involved in biological processes such as extracellular matrix organization, integrin binding, and the IL17 signaling pathway (**Figures 4E, F**).

**Figure 4:**
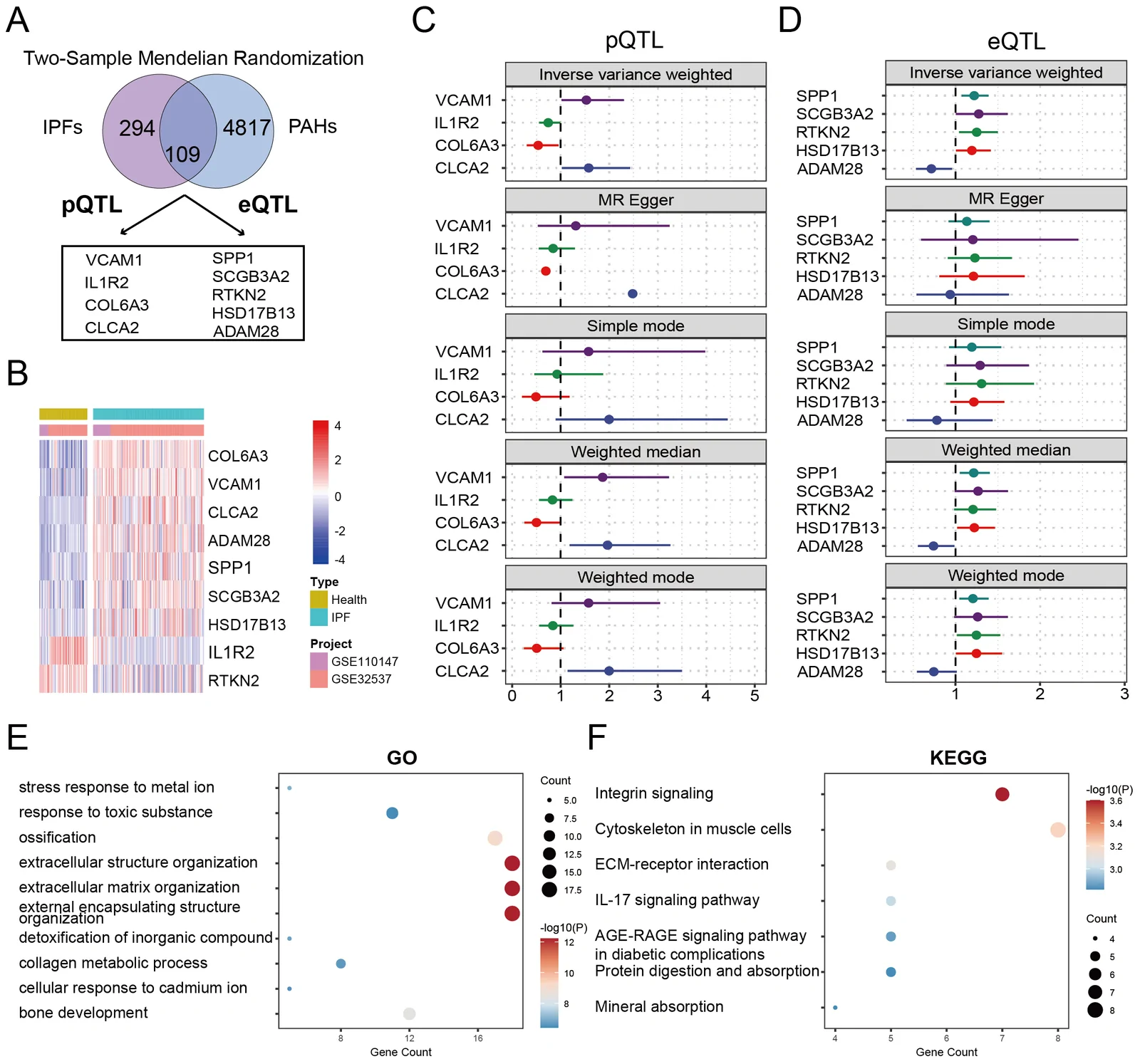
Screening of shared targets between PAHs and IPF via Mendelian randomization and functional enrichment analysis. **(A)** Venn diagram illustrating the intersection of the 403 IPF-related genes and PAH-related target genes from the CTD database. **(B)** Results of the two-sample Mendelian randomization (MR) analysis based on pQTL data. **(C)** Results of the MR analysis based on eQTL data. **(D)** Heatmap showing the expression differences of the target genes screened by MR between the control and IPF groups. **(E)** Results of GO functional enrichment analysis for the 109 overlapping genes. **(F)** Results of KEGG pathway enrichment analysis for the 109 overlapping genes.

### 3.5 Construction of IPF diagnostic and prognostic models

Based on the aforementioned 9 target genes, we constructed an IPF prognostic risk model using LASSO regression (**Figure 5A**). Kaplan-Meier survival analysis revealed that patients with high-risk scores had significantly poorer prognoses, a finding confirmed in both the training (**Figure 5B**) and validation (**Figure 5C**) sets. To validate the independent prognostic value of the risk score, multivariate Cox regression analysis was conducted, adjusting for GAP stage (**Supplementary Figure 1**). Further analysis demonstrated that IL1R2 (**Figure 5D**), SCGB3A2 (**Figure 5E**), and SPP1 (**Figure 5F**) could independently predict patient survival outcomes. Evaluation via ROC curves indicated that the prognostic model possessed excellent predictive performance (**Figure 5G**), with the AUC values for 1- and 3-year survival prediction in the validation set reaching 0.83 and 0.88, respectively (**Figure 5H**). Regarding diagnostic performance, the LASSO+XGBoost diagnostic model constructed using IL1R2, SCGB3A2, and SPP1 exhibited high robustness across 6 IPF cohorts, achieving an average AUC of 0.917 (**Figure 5I**). Additionally, the evaluation of single-gene diagnostic performance revealed that SPP1 held high diagnostic value, with an average AUC of 0.90 for IPF diagnosis (**Figure 5J**).

**Figure 5:**
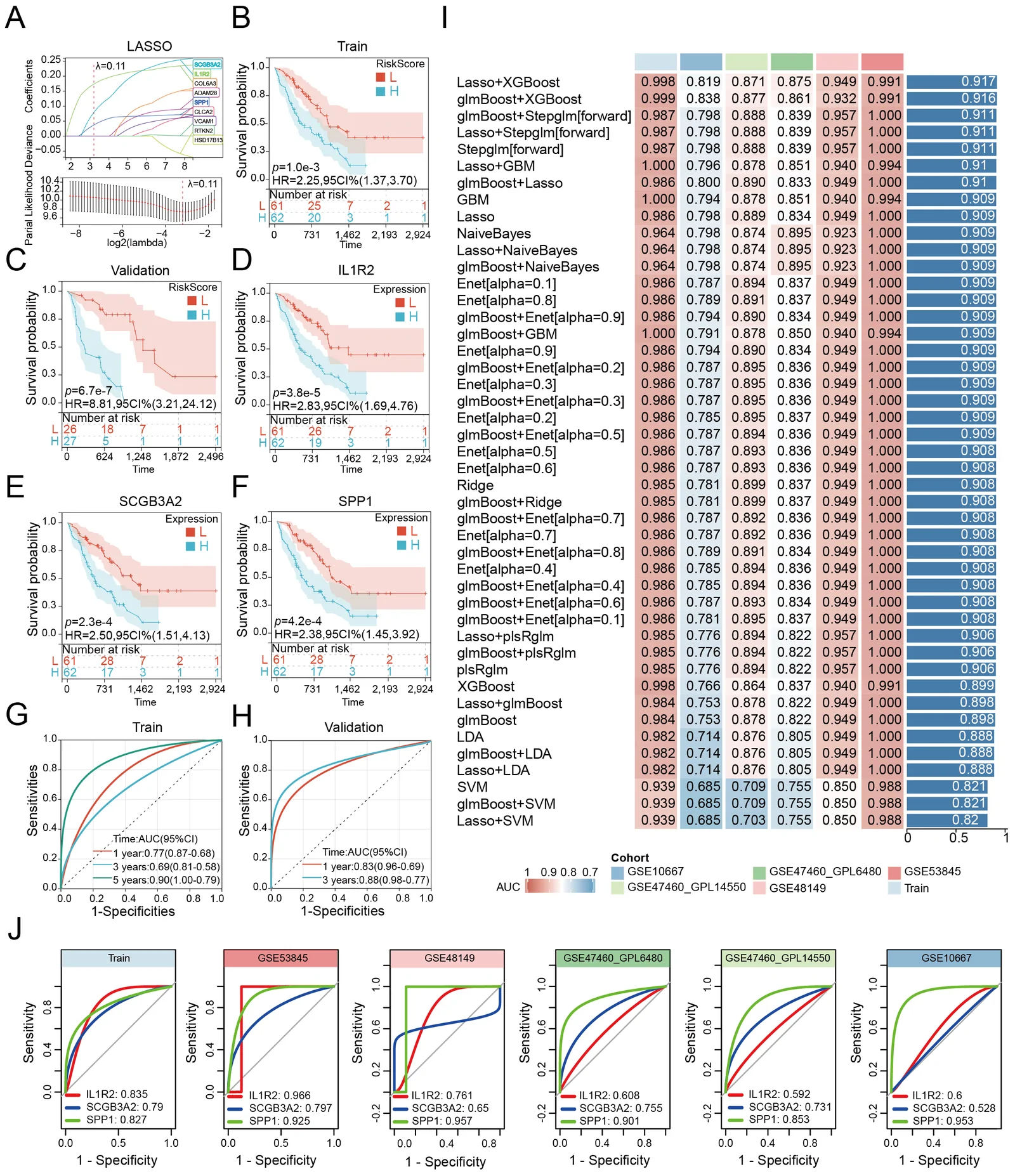
Construction and performance evaluation of IPF diagnostic and prognostic models. **(A)** Construction of the IPF prognostic risk model using LASSO regression based on 9 target genes. The GSE70866 dataset was randomly divided into a training set (n = 123) and a validation set (n = 53) at a ratio of 7:3. **(B, C)** Kaplan-Meier survival curves for patients with high- and low-risk scores in the training **(B)** and validation **(C)** sets. **(D–F)** Independent prognostic survival analyses for IL1R2 **(D)**, SCGB3A2 **(E)**, and SPP1 **(F)**, grouped by median expression levels. **(G)** Time-dependent ROC curves for predicting 1-, 3-, and 5-year prognosis in the training set. **(H)** Time-dependent ROC curves for predicting 1- and 3-year prognosis in the validation set. **(I)** Performance evaluation of the machine learning diagnostic model based on IL1R2, SCGB3A2, and SPP1 across multiple IPF cohorts. Merged and batch-corrected data from GSE32537 (50 CON, 119 IPF) and GSE110147 (11 CON, 22 IPF) were used as the training set. The validation sets included GSE10667 (15 CON, 31 IPF), GSE47460_GPL14550 (91 CON, 122 IPF), GSE47460_GPL6480 (17 CON, 38 IPF), GSE48149 (9 CON, 13 IPF), and GSE53845 (8 CON, 40 IPF). **(J)** ROC curves for the single-gene diagnosis of IPF using IL1R2, SCGB3A2, and SPP1.

### 3.6 Single-cell expression profiling of IL1R2, SCGB3A2, and SPP1

To investigate the expression characteristics of the core targets at the cellular level, we performed dimensionality reduction and clustering on the single-cell transcriptomic data from IPF and control samples, identifying five major cell populations: epithelial cells, endothelial cells, lymphoid cells, myeloid cells, and stromal cells (**Figure 6A**). **Figure 6B** illustrates the changes in the proportional distribution of each cell subpopulation between the control and IPF groups. Dot plot analysis revealed that IL1R2 was predominantly expressed in dendritic cells and macrophages, SPP1 was predominantly expressed in macrophages, and SCGB3A2 was specifically enriched in Club cells (**Figure 6C**). Differential expression analysis further indicated that IL1R2 exhibited a significant downregulation trend in the IPF group, whereas the expression of SPP1 and SCGB3A2 was significantly upregulated (**Figure 6D**).

**Figure 6:**
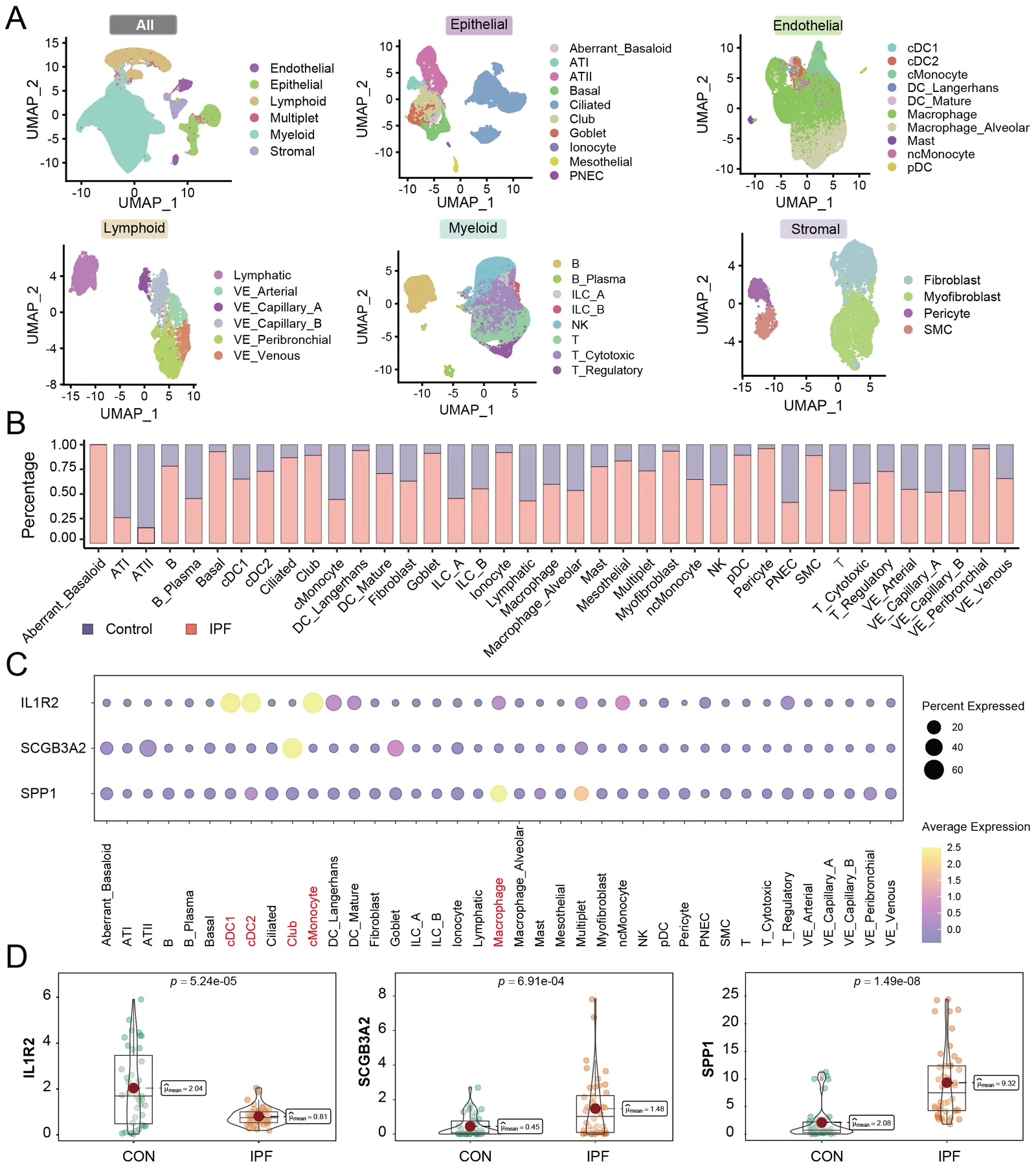
Expression patterns and cellular distribution of core targets in the IPF single-cell transcriptome. **(A)** UMAP plot of dimensionality reduction and clustering for the IPF single-cell transcriptome, showing five major cell populations. **(B)** Bar chart showing the proportional distribution of cell subpopulations between the control and IPF groups. **(C)** Expression distribution of IL1R2, SPP1, and SCGB3A2 across different cell subpopulations. **(D)** Comparison of the expression differences of IL1R2, SPP1, and SCGB3A2 between the control and IPF groups.

### 3.7 Molecular docking analysis of PAHs and IL1R2

Molecular docking between 16 PAHs and the IL1R2 protein was performed using AutoDock Vina software, and representative 3D and 2D interaction diagrams were generated (**Figure 7A**). The results indicated that PAHs primarily bind to residues TYR(B:249) and VAL(B:224) via hydrophobic interactions such as Pi-Alkyl and Alkyl, and form stable conformations with LEU(C:319) through Pi-Pi stacking. Binding energy analysis revealed that dibenz[a,h]anthracene (DBA) exhibited the lowest binding energy with IL1R2, demonstrating the strongest affinity, whereas naphthalene (NAP) had a relatively higher binding energy. Nevertheless, the binding energies of all PAHs were below -6 kcal/mol, indicating that they all possess strong binding potential. To further verify the reliability of the docking results, we introduced DeepMice, a deep learning-based binding affinity prediction tool, for independent scoring. Correlation analysis demonstrated a strong consistency between the AutoDock Vina binding energies and the DeepMice scores (i.e., lower Vina binding energies corresponded to lower DeepMice scores). Together, these findings corroborate the tight binding and conformational stability between PAHs and IL1R2 (**Figure 7B**).

**Figure 7:**
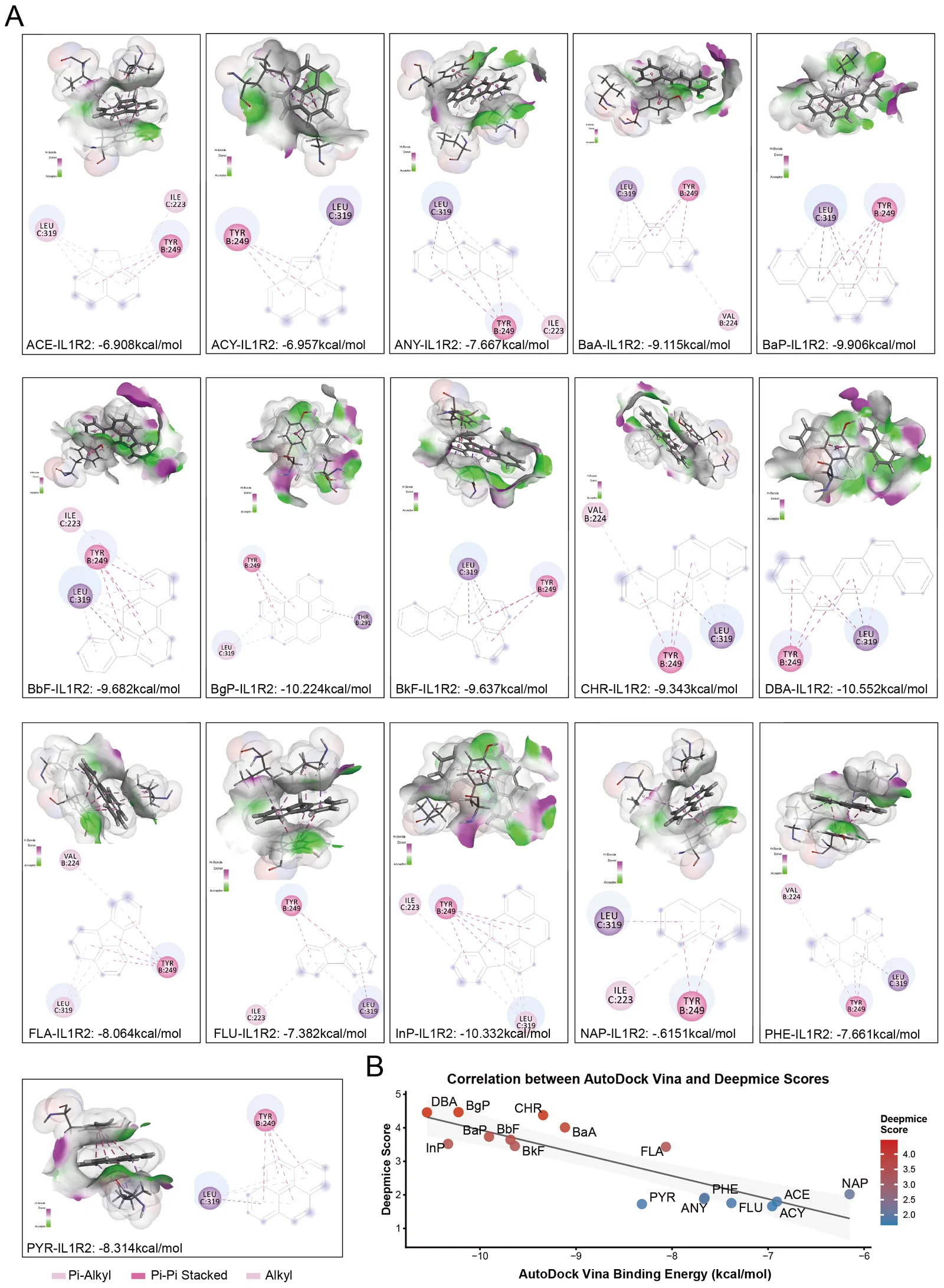
Molecular docking and affinity validation of PAHs with IL1R2. **(A)** Representative 3D and 2D interaction diagrams illustrating the binding modes of PAHs with key residues of the IL1R2 protein. **(B)** Correlation analysis between AutoDock Vina binding energies and DeepMice prediction scores, validating the tight binding and conformational stability between PAHs and IL1R2.

### 3.8 Molecular dynamics simulation analysis of the DBA-IL1R2 complex

To evaluate the dynamic binding process and structural stability of the DBA-IL1R2 receptor complex, a 100 ns molecular dynamics (MD) simulation was performed. The root mean square deviation (RMSD) of the overall backbone indicated that the complex rapidly stabilized after a brief fluctuation, suggesting that the receptor reached an equilibrium state upon DBA binding (**Figure 8A**). The radius of gyration (Rg) for each chain (A, B, and C) of the IL1R2 receptor remained stable throughout the simulation, indicating that the receptor maintained a compact three-dimensional structure upon DBA binding, without obvious structural loosening or unfolding (**Figure 8B**). Root mean square fluctuation (RMSF) analysis further illustrated the local flexibility characteristics of the residues in each chain (**Figure 8C**). The RMSD curve of the DBA ligand over time demonstrated that it maintained a stable conformation within the binding pocket (**Figure 8D**). Analysis of the minimum atomic distance between the ligand and receptor revealed that the distance was consistently maintained within a low range without abrupt changes, indicating that DBA remained stably bound within the pocket throughout the simulation without dissociation or drift (**Figure 8E**). Additionally, the solvent-accessible surface area (SASA) curve exhibited steady fluctuations, indicating that the hydrophobic core of the protein was not abnormally exposed and that the complex maintained a stably folded conformation in the solvent (**Figure 8F**). Finally, the PCA-based Gibbs free energy landscape revealed that the DBA-IL1R2 system primarily sampled conformations in the low-energy region, confirming a dominant and stable binding state (**Figure 8G**). Comparative simulations further contextualized this stability: similar to DBA, InP maintained stable binding within the pocket with converged RMSDs, whereas BgP exhibited continuous RMSD fluctuations, indicating unstable binding and shifting (**Supplementary Figure 2**). Together, these dynamics data demonstrate that the IL1R2-DBA complex maintains strong structural stability and persistent binding capacity under physiological conditions.

**Figure 8:**
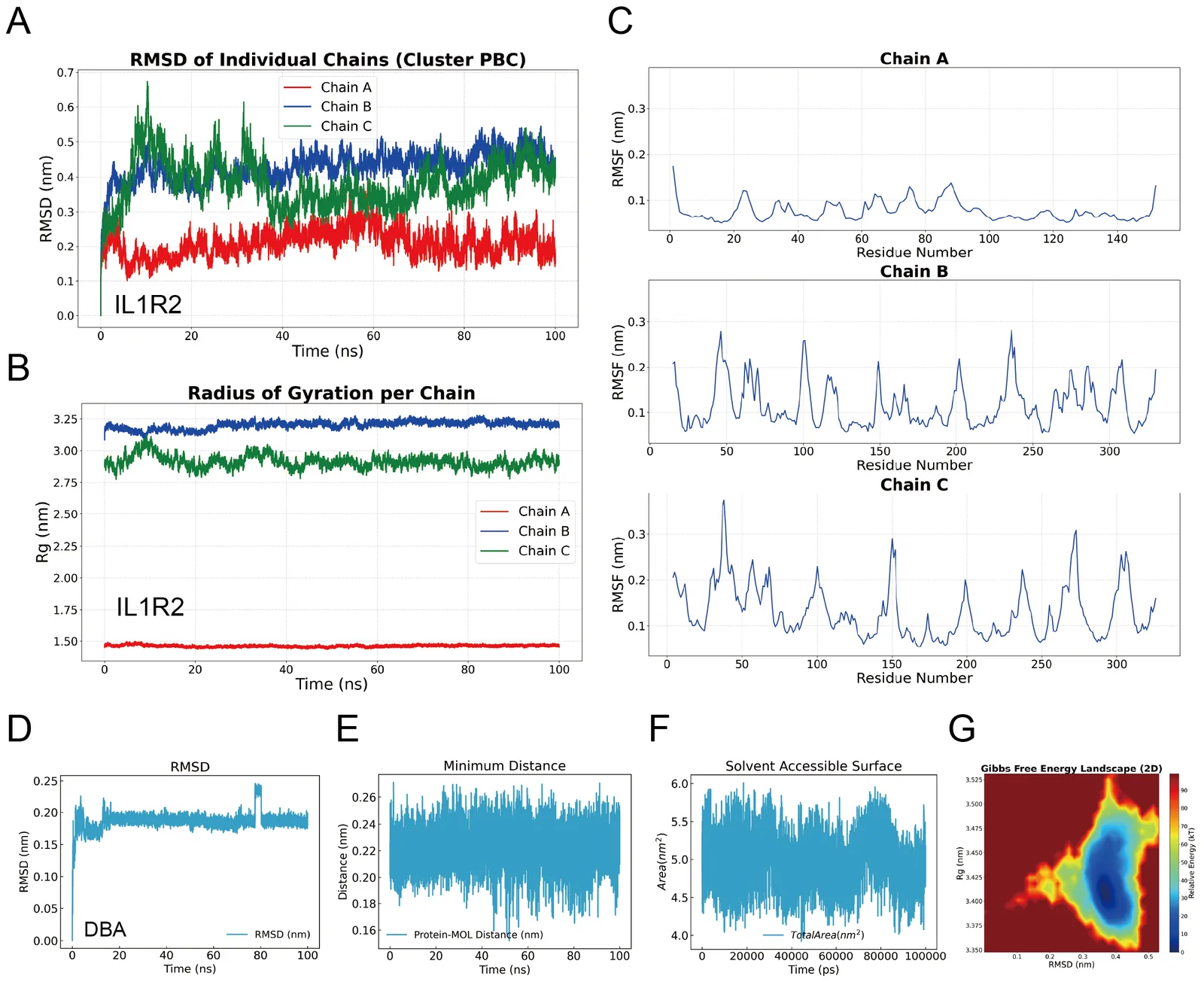
Molecular dynamics (MD) simulation and conformational stability analysis of the DBA-IL1R2 receptor complex. **(A)** Root mean square deviation (RMSD) of the IL1R2 receptor backbone over time. This reflects the deviation of the receptor’s overall conformation upon DBA binding. The curve stabilizes after a brief fluctuation, indicating that the complex reached conformational equilibrium during the simulation. **(B)** Radius of gyration (Rg) of the three IL1R2 receptor chains (A, B, and C) over time. **(C)** Root mean square fluctuation (RMSF) distribution of residues in each IL1R2 receptor chain (A, B, and C). **(D)** Root mean square deviation (RMSD) of DBA over time. **(E)** Minimum atomic distance between DBA and the IL1R2 receptor over time. This monitors the closest distance between ligand and receptor atoms; the minimum distance was consistently maintained within a low range without abrupt changes, indicating that DBA remained stably bound within the pocket during the simulation without dissociation. **(F)** Solvent-accessible surface area (SASA) of the IL1R2-DBA complex over time. This reflects the contact area between the complex and the solvent. The curve exhibits steady fluctuations, indicating that the hydrophobic core of the protein was not abnormally exposed and that the complex maintained a stably folded conformation in the solvent. **(G)** Gibbs free energy landscape based on principal component analysis (PC1 and PC2). The color gradient represents the free energy distribution across the conformational space. The dark blue low-energy region corresponds to the most stable binding conformation of the system, reflecting the dominant conformational sampling space of DBA within the IL1R2 pocket.

### 3.9 Loss of IL1R2 drives spatial paracrine communication between macrophages and fibroblasts

IL1R2 is predominantly expressed in myeloid cell subpopulations but is significantly downregulated in IPF tissues. Compared to the control group, the proportions of IL1R2-positive DCs, macrophages, and monocytes were significantly decreased in the IPF group (**Figures 9A, B**). In IPF macrophages (including alveolar macrophages), IL1R2 expression levels exhibited a significant negative correlation with IL1 signaling pathway intensity. This suggests that IL1R2 acts as a decoy receptor exerting negative regulatory effects, and its loss leads to the abnormal activation of IL1 signaling within macrophages (**Figure 9C**). Meanwhile, the expression of IL1R2 is closely related to the progression of IPF. By analyzing the expression of IL1R2 across different Gender, Age, Physiology (GAP) stages and its correlation with IL-1β in the GSE32537 cohort, we found that IL1R2 is upregulated in patients with advanced IPF, which might be associated with a compensatory response against the overactivated IL-1 signaling pathway (**Supplementary Figure 3**). Conversely, in fibroblasts, IL1 signaling intensity showed a significant positive correlation with fibrosis scores, indicating that the abnormally enhanced IL1 signaling directly drives the pro-fibrotic phenotype of target cells (**Figure 9D**). Spatial transcriptomic data further confirmed the physical basis for this interaction: numerous spatial spots exhibiting tight co-localization between IL1R2-expressing macrophages and fibroblasts were present *in situ* within IPF tissues, providing a spatial structural foundation for paracrine signal transmission (**Figure 9E**). To provide direct functional evidence for this crosstalk, THP-1-derived macrophages treated with DBA or INP were co-cultured with MRC-5 fibroblasts, resulting in a significant upregulation of the fibroblast activation marker α-SMA in MRC-5 cells (**Figure 9F**). Furthermore, CellChat analysis revealed that, acting as the signal source, IL1R2-expressing macrophages transmitted significantly enhanced IL1 signaling intensity and interaction weights to fibroblasts and myofibroblasts (**Figure 9G**). In summary, the downregulation of IL1R2 in the IPF microenvironment removes the “brake” mechanism on IL1 signaling in macrophages. These macrophages closely interact with fibroblasts in space, driving the fibrotic process through enhanced IL1 paracrine signaling.

**Figure 9:**
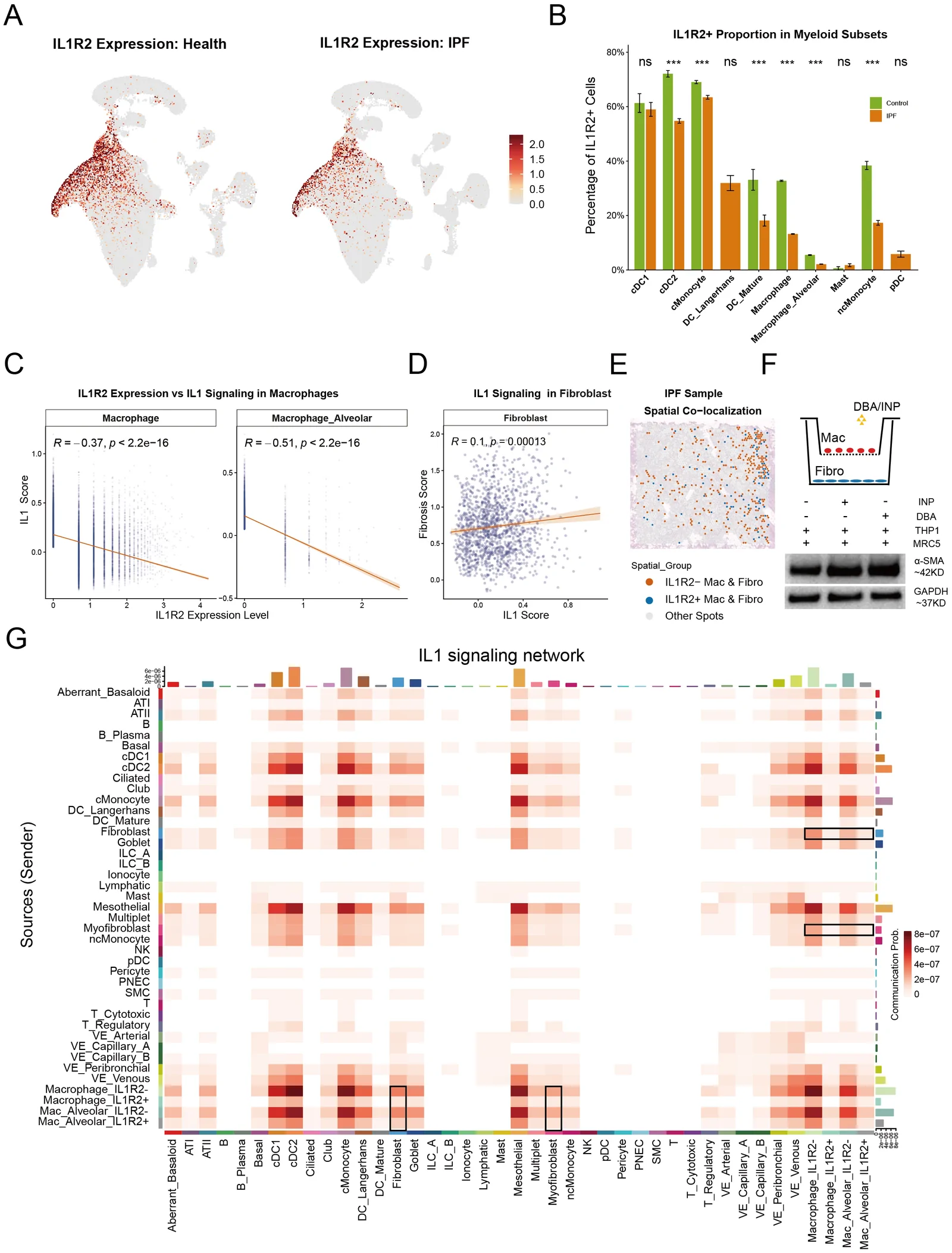
Loss of IL1R2 drives pro-fibrotic phenotypic transformation and spatial communication in macrophages. **(A)** UMAP plots showing the expression distribution of IL1R2 in single cells from control and IPF patients, with red indicating high expression. **(B)** Bar chart comparing the proportions of IL1R2-positive cells across different myeloid subpopulations between the control and IPF groups (***P < 0.001; ns, not significant). **(C)** Scatter plots showing the correlation between IL1 signaling pathway activity and IL1R2 expression levels in macrophages and alveolar macrophages from IPF patients. **(D)** Scatter plot showing the correlation between IL1 signaling pathway activity and fibrosis scores in fibroblasts from IPF patients. **(E)** Spatial transcriptomic data from IPF patients showing the co-localization distribution of IL1R2-expressing macrophages and fibroblasts within tightly connected spatial spots. **(F)** THP-1-derived macrophages treated with DBA or INP were co-cultured with MRC-5 fibroblasts. The expression of α-SMA, a fibroblast activation marker, was detected in MRC-5 cells. **(G)** Heatmap from CellChat analysis illustrating the communication intensity and interaction weights of IL1 signaling between IL1R2-expressing macrophages (senders) and fibroblasts/myofibroblasts (receivers). Black boxes highlight the IL1R2-positive and IL1R2-negative sender macrophage subpopulations, as well as the receiver fibroblasts and myeloid-derived fibroblasts.

## 4 Discussion

In this study, we integrated network toxicology with multi-omics data to systematically analyze the interactions between 16 PAHs and IPF target proteins. Through the intersection of the WGCNA co-expression network and differentially expressed genes, 109 overlapping genes were identified. Subsequent two-sample Mendelian randomization (MR) analysis further pinpointed three core molecules: SPP1, IL1R2, and SCGB3A2. The pathological progression of IPF is inherently a dynamic process characterized by multicellular interactions and signaling pathway dysregulation. These three molecules directly target the crux of the disease mechanisms: SPP1 drives pro-fibrotic immune responses (33), IL1R2 reflects an imbalance in inflammatory regulation (34), and SCGB3A2 abnormalities indicate impaired epithelial protective functions (35). Subsequent molecular docking, molecular dynamics (MD) simulations, and single-cell spatial transcriptomic analyses further confirmed that upon PAH exposure, the IL1R2 target is not only significantly dysregulated in its expression but also acts as a key force driving the remodeling of the fibrotic microenvironment.

SPP1 has been identified as a central driver and key biomarker in the progression of IPF. In this study, MR analysis confirmed a causal link between SPP1 and IPF, demonstrating its excellent diagnostic performance (mean AUC of 0.9). Single-cell analysis further revealed that SPP1 is predominantly expressed in specific macrophage subsets. Previous studies indicate that SPP1-expressing macrophages accumulate heavily within the alveolar spaces during early IPF stages, closely correlating with fibroblast activation (36). Beyond directly promoting pulmonary fibrosis via the PI3K/Akt/mTOR pathway, SPP1 also accelerates disease progression by inducing epithelial-mesenchymal transition (EMT) (37). Furthermore, SPP1 promotes M2 macrophage polarization via the Jak2/Stat3 pathway, thereby fostering a pro-fibrotic immune microenvironment (36). Similarly, in systemic sclerosis-associated interstitial lung disease, SPP1+ macrophages have been proven to act as key effector cells driving fibrosis (38, 39). Clinically, SPP1 levels in serum or bronchoalveolar lavage fluid correlate significantly with disease severity and prognosis in IPF patients (40–42), which aligns with the high-risk prognostic profile observed in our study.

SCGB3A2 expression is significantly upregulated in IPF, and MR analysis (IVW > 1) reveals a positive causal link to IPF risk, where elevated expression portends a poorer prognosis. The core pathogenesis of IPF involves sustained injury and aberrant repair of alveolar epithelial cells (43); however, single-cell transcriptomic mapping in our study reveals that SCGB3A2 is specifically enriched in Club cells. As an airway secretory protein, its aberrant upregulation likely reflects the phenotypic remodeling and dysfunction of injured epithelial cells within a pathological microenvironment (44). Integrating our causal inference and survival analyses, we posit that SCGB3A2 is not merely an epiphenomenon; rather, it likely drives the fibrotic cascade directly by mediating EMT or activating fibroblasts (45). This triad of high expression, high risk, and poor prognosis suggests that SCGB3A2 holds promise not only as a novel biomarker for prognostic evaluation in IPF but also as a compelling therapeutic target for antifibrotic intervention.

IL1R2 plays a surprisingly complex role in IPF. Although its overall expression is downregulated in IPF tissues, survival analysis shows that higher levels actually correlate with worse outcomes. This apparent paradox may reflect a dynamic shift in immune regulation across different stages of the disease. As a decoy receptor, IL1R2 binds IL-1β without triggering downstream signals, effectively putting the brakes on inflammation (42). Early in the disease, a drop in IL1R2 removes this brake, allowing inflammation to spiral out of control following initial insults (such as PAH-induced epithelial damage) (46, 47). However, as IPF progresses, IL1R2 shows an upregulation trend. This is likely a response to the overwhelming IL-1β-driven signaling in the late-stage microenvironment. Crucially, this upregulation fails to halt the fibrotic progression, which may explain why higher IL1R2 levels paradoxically accompany poorer outcomes. Furthermore, molecular docking and 100-ns MD simulations show that PAHs, typified by DBA, can stably lodge themselves inside IL1R2’s binding pocket. This suggests that PAH exposure might directly block or allosterically distort the receptor’s ligand-binding domain, further crippling IL1R2’s ability to neutralize IL-1β.

More importantly, by integrating single-cell and spatial transcriptomic data, our study uncovers the potential mechanisms through which aberrant IL1R2 expression drives disease progression. Within the IPF microenvironment, IL1R2 is predominantly expressed in myeloid cells. Its downregulation effectively releases the “brakes” on the IL-1 signaling pathway within macrophages, triggering its aberrant activation. Spatial transcriptomic data visually demonstrate numerous regions *in situ* within IPF tissues where IL1R2-negative macrophages and fibroblasts are tightly co-localized. CellChat analysis further corroborates that these IL1R2-negative macrophages act as signaling hubs, transmitting significantly amplified IL-1 signals to fibroblasts and myofibroblasts. This signal intensity positively correlates with the fibrotic scores of target cells, thereby directly propelling their phenotypic transition. However, bulk RNA-level prognostic analyses indicate that patients with higher IL1R2 expression suffer poorer outcomes. This apparent paradox actually reflects the dynamic evolution of the disease across different stages. During early fibrogenesis, the loss of IL1R2 dominates the aberrant paracrine interplay between macrophages and fibroblasts. Conversely, in advanced IPF, confronted with a fierce and unchecked inflammatory storm, specific cell subpopulations may compensatorily upregulate IL1R2 in an attempt to curb the overactivated IL-1 signaling (48). Here, its elevated expression is not a pathogenic driver, but rather an end-stage marker of an exceedingly high local inflammatory burden. Consequently, IL1R2 plays a dynamic role throughout disease evolution. Future therapeutic strategies targeting this molecule should not be confined to blanket inhibition or activation; rather, they require precise modulation tailored to the patient’s molecular subtyping and specific disease stage.

Although this study sheds light on the toxicological mechanisms of PAH-induced IPF, several limitations should be acknowledged. Mechanistically, our findings are primarily grounded in *in vitro* assays and computational modeling, lacking *in vivo* rescue evidence to solidify the causal claims. Specifically, while MR analysis established a causal link between gene/protein levels and IPF risk, the PAH-to-gene perturbation step relies on the CTD database, rendering the full causal chain largely inferential. Similarly, although our co-culture model confirmed that PAH-exposed macrophages activate fibroblasts, the use of unfractionated macrophages precludes us from definitively attributing this effect solely to the IL1R2-negative subset. Furthermore, functional validation was restricted to four representative PAHs; thus, extrapolating these findings to all 16 priority-controlled PAHs requires caution. Finally, the absence of smoking data in our retrospective cohort limits robust confounding adjustment, leaving genetic susceptibility and environmental co-exposures as additional unmeasured variables.

To address these limitations, future mechanistic work should focus on macrophage subpopulation sorting and *in vivo* models. This will help confirm the specific role of IL1R2-negative macrophages and bridge the current gaps in the PAH-gene-IPF causal chain. Testing a wider range of PAHs in these models will also show if our findings can be generalized. Clinically, prospective cohorts with detailed exposomics and smoking data are needed to clarify dose-response relationships and properly adjust for confounders. Ultimately, applying these findings to tighten PAH emission controls and set health-based environmental benchmarks is key to reducing population exposure and easing the IPF burden at its source.

## 5 Conclusion

By integrating network toxicology, multi-omics, and computational chemistry, this study investigated the toxicological mechanisms underlying idiopathic pulmonary fibrosis (IPF) induced by 16 polycyclic aromatic hydrocarbons (PAHs). We identified key targets such as SPP1, IL1R2, and SCGB3A2, and validated the high-affinity, stable binding between PAHs (particularly DBA) and IL1R2. Subsequent single-cell and spatial transcriptomic analyses demonstrated that IL1R2-negative macrophages, by releasing the “brakes” on IL-1 signaling, tightly co-localize with fibroblasts and amplify paracrine communication, thereby driving the establishment of a pro-fibrotic microenvironment. These findings enrich the molecular and cellular understanding of PAH-induced pulmonary fibrosis and provide a reference framework for the risk assessment and subsequent therapeutic intervention of environmental pollutant-associated IPF.

## Data Availability

The original contributions presented in the study are included in the article/**Supplementary Material**. Further inquiries can be directed to the corresponding authors.
