# Supplementary material for: Integrating multi-omics and machine learning to reveal IL1R2-driven macrophage-fibroblast spatial interactions in polycyclic aromatic hydrocarbon-induced pulmonary fibrosis
Source: Front Immunol. 2026 Oct 21;17:1968775. doi: 10.3389/fimmu.2026.1968775 (PMC13730967; doi:10.3389/fimmu.2026.1968775)
Supplement: Supplementary file 1 [file Table2.docx]

**Supplementary Figures**

**Supplementary Figure 1. Multivariate Cox regression analysis of the risk score and IPF clinical variables.** (A) Risk score, age, and gender. (B) Risk score and GAP.

**Supplementary Figure 2. Molecular dynamics (MD) simulation results for InP-IL1R2 and BgP-IL1R2 complexes.** (A) InP-IL1R2 complex. Time evolution of radius of gyration (Rg), IL1R2 backbone RMSD, InP positional RMSD, solvent accessible surface area (SASA), and minimum atomic distance (MAD) over the 100 ns simulation. The two-dimensional free energy landscape (FEL) is constructed using RMSD and Rg as reaction coordinates. (B) BgP-IL1R2 complex. Time evolution of Rg, IL1R2 backbone RMSD, BgP positional RMSD, SASA, and MAD. The FEL is constructed as in (A).

**Supplementary Figure 3. Stage-dependent IL1R2 expression and its correlation with IL-1β in the GSE32537 cohort.** (A) Box plots showing IL1R2 expression levels in Normal Controls and IPF patients stratified by GAP stages (I, II, and III). (B) Scatter plot showing the correlation between IL1R2 and IL-1β (IL1B) expression levels in IPF patients.  Data are derived from the GSE32537 cohort.
